# Fifth Annual Report of Trustees of Westborough Insane Hospital

**Published:** 1890-02

**Authors:** 


THE FIFTH ANNUAL REPORT OF THE TRUSTEES OF THE WESTBOROUGH (MASS.) INSANE HOSPITAL, FOR THE YEAR ENDING SEPT. 30TH, 1889,

Comes to us and shows that Homœopathy need not fear to be placed on trial in the treatment of the insane. The leaven is at work, and the right usually triumphs sooner or later.

